# Predictors of public and private healthcare utilization and associated health system responsiveness among older adults in Ghana

**DOI:** 10.1080/16549716.2017.1301723

**Published:** 2017-06-04

**Authors:** Mamaru Ayenew Awoke, Joel Negin, Jette Moller, Penny Farell, Alfred E. Yawson, Richard Berko Biritwum, Paul Kowal

**Affiliations:** ^a^ Department of Public Health Sciences, Karolinska Institutet, Stockholm, Sweden; ^b^ Monitoring, Evaluation and Research Unit, Amref Health Africa, Addis Ababa, Ethiopia; ^c^ School of Public Health, University of Sydney, Sydney, Australia; ^d^ Department of Community Health, University of Ghana, Accra, Ghana; ^e^ Research Centre for Gender, Health & Ageing, University of Newcastle, Newcastle, Australia; ^f^ SAGE, World Health Organization, Geneva, Switzerland

**Keywords:** Healthcare utilization, health system responsiveness, older adults, WHO-SAGE, Ghana

## Abstract

**Background**: Previous studies investigating factors associated with healthcare utilization by older Ghanaians lack distinction between public and private health services. The present study examined factors associated with public and private healthcare service use, and the resulting perceived health system responsiveness.

**Objectives**: To identify factors associated with public and private healthcare utilization among older adults aged 50 and older in Ghana; and to compare perceived differences in health system responsiveness between the private and public sectors.

**Methods**: Cross-sectional data was analyzed from the World Health Organization Study on global AGEing and adult health (SAGE) Wave 1 in Ghana. Using Andersen’s conceptual framework, public and private outpatient care utilization was examined using multinomial logistic regression to estimate and identify predictor variables associated with the type of outpatient healthcare facility accessed. Health system responsiveness was compared using chi-square tests.

**Results**: Of 2517 respondents who used outpatient care in the 12 months preceding interview, 51.7% of respondents used a public facility, 17.8% a private facility, and 30.5% used other facilities. Older age group, higher education and higher wealth were associated with the use of private outpatient healthcare services. Using public outpatient care facilities was associated with having health insurance. Respondents with two or more chronic conditions were more likely to use public and private outpatient care than other facilities. Perceived health system responsiveness was better in private for-profit than in public and private not-for-profit healthcare facilities.

**Conclusions**: This study suggested that higher wealth and multimorbidity were significant predictors of public and private outpatient healthcare utilization; however, health insurance was a predictor only for the use of public facilities. Future mixed-method studies could further elucidate factors influencing the choice of public and private outpatient healthcare use.

## Background

In 2016, the global number of adults aged 60 years and older was 900 million and this is projected to be more than 2 billion by 2050 [1]. The number of older adults is already high and increasing in low- and middle-income countries (LMICs) [2–4]. According to demographic projections, the number of adults aged 60-plus years in sub-Saharan Africa will increase from 46 million (4.8% of the total population) in 2015 to 161 million by 2050 (7.5% of the total population) [4]. The proportions in Ghana are higher than the regional average, with 5.3% of the current total population aged 60-plus years, reaching 9.7% by 2050 [5].

The growth of the older population in African countries will likely put pressure on healthcare delivery systems. As people age, they are more likely to need healthcare due to declining functional capacity and the increased likelihood of having more complex health problems. Older adults who have multiple chronic conditions are known to have higher healthcare use [6,7]. Prior studies have reported the factors associated with healthcare utilization by older adults [8–10], including having multiple chronic conditions [11–13], high level of education [13], living alone, poor self-perceived health [14], older age, higher income, access to health insurance [12,15] and urban residence [16].

The foundations of Universal Health Coverage are grounded in public healthcare systems, yet private healthcare services play a crucial role in contributing to healthcare delivery in many lower-income countries [17], and are expanding in sub-Saharan Africa [18]. Examining factors associated with the type of outpatient healthcare provider among older adults may help to improve health service delivery and health system governance. However, differences between public and private healthcare utilization by older adults have not been well documented. Further, healthcare delivery in Ghana is a mix of private for-profit, private non-profit, and public healthcare facilities; and traditional healthcare providers. Given this mixture of providers in Ghana’s health system, and high utilization of charity-based and traditional healthcare [19], little is known about what influences the type of healthcare used by older Ghanaian adults. Public health facilities are operated by the government, and deliver the largest proportion of healthcare services in Ghana. Private healthcare consists of private for-profit facilities owned by private individuals or companies which are privately funded through payments for medical services, and private not-for-profit services such as mission or faith-based facilities involved in the direct delivery of health services [19]. The availability and ownership of healthcare facilities across the country have been reported to include 1607 governmental facilities, 1277 private for-profit facilities, 245 mission and 91 quasi-governmental facilities [20].

In Ghana, out-of-pocket (‘cash and carry’) payments account for the single largest share (45%) of total healthcare financing [21,22]. Thus, the ‘cash and carry’ system of paying for healthcare at the point of service remains one of the barriers to healthcare access. Ghana implemented a National Health Insurance Scheme (NHIS) in 2003 which became operational in most facilities in 2005 to protect the people of Ghana against catastrophic health expenditures, aiming to promote universal coverage and equity in healthcare delivery services. However, it has also been reported that the NHIS contribution from the formal employment sector is very regressive, in that people with lower income contribute a higher share of their income than do people with higher income [21]. Coverage also remains low, with only 34% of people being active members (valid cardholder members) of the NHIS [23]. Furthermore, it is mainly public and mission-based healthcare facilities which provide healthcare through the NHIS; most private for-profit facilities require payment at the point of use. It has also been reported that the total benefits from private and public services in Ghana tend to benefit richer more than poorer individuals [21].

One way to assess how well a health system is performing, is to ask about the experiences of those who interact with the system, in this case, asking the opinions of older Ghanaians who used outpatient healthcare. One method is to simply ask about patient satisfaction. For instance, a study in Ghana reported high perceived quality of care and satisfaction among patients interviewed after visiting healthcare facilities [24]. In contrast, a study in South Africa found that a high percentage of adults reported dissatisfaction in all types of healthcare facilities [25]. However, these studies do not address health system responsiveness differences between public and private healthcare facilities.

The World Health Organization (WHO) developed health system responsiveness as a concept that documents what actually transpires when individuals come into contact with the health system and the environment in which they were treated [26,27]. The economic impacts on societies that are increasingly concerned about the needs of older adults may be mitigated by a highly responsive healthcare system through improved health outcomes and cost-efficiencies. Responsiveness is one mechanism for monitoring how well the healthcare system may adapt to future population health profiles. Although limited studies exist in sub-Saharan Africa, a study in South Africa found that health system responsiveness was lower in public healthcare facilities compared to private facilities [28].

Understanding, from the patient’s perspective, factors influencing the type of public and private healthcare facilities used and how well the system responds to their care needs provides information that could help to improve healthcare service delivery. The current study aimed to identify factors associated with the use of public and private outpatient healthcare among older adults aged 50 and older in Ghana. The study also sought to compare health system responsiveness among older adults who received public or private outpatient healthcare services.

Findings from this study can provide useful information to health planners and policy-makers for the appropriate planning of the healthcare system structure, policies and programs for public and private health services, and to healthcare providers regarding how healthcare services need to be organized and reformed to meet the needs of older adults.

## Methods

### Data source

The WHO Study on global AGEing and adult health (SAGE) Wave 1 was implemented in six LMICs: China, Ghana, India, Mexico, Russian Federation and South Africa, during 2007–2010 [29]. SAGE Ghana Wave 1 took place during 2007/2008. Data for this study therefore was obtained from the SAGE Wave 1 survey in Ghana.

### Data collection and sampling procedures

In the SAGE Wave 1 survey in Ghana a stratified multistage cluster sampling method was used to collect data from a nationally representative sample of adults aged 50 years and older, plus a smaller sample of adults aged 18–49 years, using standardized survey instruments. Trained interviewers conducted structured face-to-face interviews to collect information on socio-demographic characteristics, employment status and type, access to health insurance, chronic health conditions, healthcare utilization, caregiving and health system responsiveness. Further details of the sampling methods and data collection procedures used in Ghana’s SAGE have been described elsewhere [30,31].

### Study population

The sample selected for analysis from SAGE Ghana Wave 1 included community-dwelling persons aged 50 and older residing in all regions of Ghana, excluding institutionalized people (n = 4724). Of these, 4264 respondents completed the interview. This analysis included only older adults who had used outpatient care in the 12 months prior to interview. Those who did not seek care and reported ‘no outpatient care in last 12 months’ were not included in the analysis. Thus, an analytical sample size of 2517 respondents was obtained.

### Predictor variables and measures

Predictor variables were considered based on Andersen’s conceptual framework for a healthcare utilization model, which includes predisposing characteristics of the individual, enabling factors and need factors [32]. Predisposing factors include demographic characteristics such as age, sex, marital status and family size, and social structure including employment, education and ethnicity were also included as covariates. Enabling factors represent material resources such as income, health insurance and distance from health services. Need factors include severity of illness, self-rated health and multiple chronic conditions.

Regarding predisposing factors, variables included in the analysis were age, sex, marital status and educational status. Age was categorized into four groups (50–59, 60–69, 70–79 and 80+); marital status was classified as currently married/cohabitating, never married or separated/divorced/widowed; and educational level was classified as no formal education, completed primary school or less, completed secondary school, completed high school or above.

Enabling factors included in the analysis were residence (urban/rural), possession of health insurance, type of employment and wealth quintiles. The wealth measured in quintiles was derived from possession of household durable assets, dwelling characteristics (type of floor, wall materials and cooking facilities) and access to services (improved water, sanitation and cooking fuel) [31]. The lowest wealth quintile (Q_1_) represents the poorest and the highest quintile (Q_5_) represents the richest.

Two measures of Andersen’s need factors were included: the number of chronic conditions and self-rated health status were included as a third group of variables. Presence of chronic conditions was defined based on the total number of self-reported chronic conditions. In the analysis, respondents were grouped according to the number of chronic health conditions reported: ‘none’ if no chronic diseases were reported, ‘one chronic condition’ if only one chronic disease, and ‘multimorbidity’ for those with two or more chronic diseases. Overall general self-rated health (perceived health status) was assessed through the question: ‘In general, how would you rate your health today?’ by using a five-point response scale of very good, good, moderate, bad and very bad.

### Outcome variable and measure

The outcome variable was the type of outpatient care service used for the respondent’s most recent visit based on the question: ‘Over the last 12 months, did you receive any healthcare, not including an overnight stay in hospital or long-term care facility?’ A ‘yes’ response elicited further questions about the type of facility; these were categorized as: (1) private facility: private doctor, clinic or hospital; (2) public facility: public clinic or hospital; and (3) other facilities: charity clinic or hospital, homecare services or other.

### Health system responsiveness and measure

Health system responsiveness covered seven domains: (1) amount of waiting time, (2) experience of being treated respectfully, (3) clarity of information given by care providers, (4) level of involvement in decision making, (5) ability to talk privately (confidentiality), (6) ease of access and (7) cleanliness of the facility. Response categories were based on a five-point Likert-type scale (very good, good, moderate, bad and very bad) for each domain.

### Statistical analysis

The unweighted sample and weighted percentage distribution of the three health facility categories (private facility, public facility and other health facilities) were presented by each categorical predictor variable. Bivariate analyses using chi-square tests were conducted for each predictor variable. Chi-square tests were also used to compare health system responsiveness. Homecare and ‘other’ services were excluded from the analysis of health system responsiveness because questions were structured and designed to assess ambulatory care for patients who visited healthcare facilities.

Multinomial logistic regression was employed using ‘other facilities’ as the reference category to identify predictor variables associated with public and private outpatient care utilization. In the multivariate analysis, three consecutive models were developed based on Andersen’s conceptual model. In the first step (Model 1), predisposing factors (socio-demographics) such as age, sex and education were entered into the model; followed by enabling factors (residence, wealth quintiles, health insurance and type of employment) to build Model 2; and lastly, chronic health conditions and self-rated health (need factors) were included to build the final model (Model 3).

All analyses were performed using STATA SE version 14.1 (Stata Corporation, College Station, Texas, USA). The data was weighted using post-stratified individual sample probability weights based on the selection probability at each stage of selection. The sampling weights were post-stratified by region, residence (urban/rural), sex and age groups (18–49, 50–59, 60–69, 70+) according to the 2009 projected population estimates obtained from the Ghana Statistical Service [31]. The ‘svy’ command was employed in STATA to adjust for the complex survey design characteristics such as sampling weights, strata and clustering. Statistical significance was considered at *p*-value ≤ 0.05. Odd ratios (OR) with 95% confidence intervals (CI) were reported for each predictor variable.

## Results

### Type of outpatient healthcare facility used by demographic, socioeconomic and health status characteristics

Overall, of 2517 respondents who sought and accessed outpatient care in the 12 months preceding the survey, 1315 (51.7%) of respondents used a public facility, 402 (17.8%) used a private facility and 800 (30.5%) used other facility types. Table 1 presents the type of facility accessed by respondent demographic and socioeconomic characteristics, and health status. The proportion of public, private and other service providers used was highest among the 50–59-year age group. Healthcare utilization was lowest among the oldest age group (80+). No significant sex differences were observed in the types of service providers used.

**Table 1.** Distribution of demographic, socioeconomic and health characteristics, by the type of healthcare facility used, SAGE Ghana Wave 1 2007/08.

	Type of health facility							
	Private		Public		Others^a^			
Variable	n	%	n	%	n	%	F	*p*-value
***Age group***								
50–59	134	32.4	450	35.1	312	40.0	1.3	0.262
60–69	122	29.3	362	28.2	219	27.5		
70–79	108	27.8	355	26.1	168	21.0		
80+	38	10.6	148	10.6	101	11.6		
***Sex***								
Male	188	51.5	611	46.8	404	49.4	1.6	0.208
Female	214	48.5	704	53.2	396	50.6		
***Residence***								
Urban	220	52.9	623	46.8	529	67.9	8.9	< 0.001
Rural	182	47.1	692	53.2	271	32.1		
***Educational status***								
No formal education	163	35.9	683	50.1	435	53.5	4.5	< 0.001
Primary completed or less	99	26.3	268	20.9	179	21.8		
Secondary school completed	28	6.5	71	5.6	21	3.0		
High school and above	111	31.3	290	23.4	164	21.7		
***Marital status***								
Never married	3	0.8	15	1.3	10	1.2	0.9	0.441
Currently married/cohabitating	223	62.4	697	56.3	429	57.7		
Separated/widowed	174	36.9	598	42.5	360	41.2		
***Wealth quintile***								
Q1 (poorest)	48	9.1	172	12.1	170	21.2	10.5	< 0.001
Q_2_	53	15.2	233	16.6	184	21.7		
Q_3_	75	19.2	255	20.7	198	25.3		
Q_4_	84	20.0	325	24.5	138	18.1		
Q5 (richest)	140	36.6	329	26.2	109	13.7		
***Health insurance***								
Yes	185	44.1	721	53.3	255	33.2	21.6	< 0.001
No	217	56.0	594	46.7	545	66.8		
***Type of job employment***								
Public sector	39	9.9	170	14.0	53	7.3	4.1	< 0.001
Private sector	18	3.9	48	4.1	25	3.3		
Self-employed	316	81.7	991	75.1	680	85.0		
Informal employment	19	4.5	84	6.9	35	4.4		
***Chronic health conditions***								
None	89	21.3	366	27.2	244	29.1	5.7	< 0.001
One chronic condition	208	52.0	635	50.0	445	56.8		
Two or more chronic conditions	103	26.8	298	22.7	108	14.1		
***Self-rated health***								
Good/very good	172	42.4	451	35.2	265	32.7	2.8	0.030
Moderate	161	40.5	609	45.2	349	42.8		
Bad/very bad	69	17.1	255	19.6	186	24.4		

Notes: N: unweighted; % (percent): the weighted proportions.

Other services include charity clinic, charity hospital, home visits and other.

Respondents residing in urban areas had higher proportions of private and other types of outpatient care visits compared to those in rural areas: 52.9% of private facility visits and 67.9% of visits to other facilities were by those in urban areas. The proportion using private providers increased with increasing wealth quintile, and was highest among the highest wealth quintile (36.6%). On the other hand, a large proportion of users of other providers was among the lower wealth quintile groups.

### Factors influencing the choice of health facilities

As demonstrated in Model 3 of Table 2, the odds of using private facilities vs. other (non-public) types of facilities increased significantly with increasing age. A similar trend was seen with increasing educational level.

**Table 2.** Odds ratios from multinomial logistic regression models of predisposing, enabling and need factors associated with outpatient public and private healthcare utilization, SAGE Ghana Wave 1, 2007/08.

	Model 1		Model 2		Model 3	
	Private vs. others^a^	Public vs. others^a^	Private vs. others^a^	Public vs. others^a^	Private vs. others^a^	Public vs. others^a^
**Predictor**	OR (95% CIs)	OR (95% CIs)	OR (95% CIs)	OR (95% CIs)	OR (95% CIs)	OR (95% CIs)
***Age group***						
50–59	1	1	1		1	1
60–69	1.58 (1.1–2.25)	1.24 (0.94–1.65)	1.49 (1.04–2.14)	1.09 (0.81–1.46)	**1.56 (1.08–2.25)**	1.09 (0.81–1.48)
70–79	2.30 (1.43–3.7)	1.59 (1.18–2.1)	2.13 (1.3–3.5)	1.23 (0.91–1.66)	**2.17 (1.34–3.52)**	1.24 (0.91–1.69)
80+	1.91 (1.07–3.38)	1.24 (0.84–1.83)	1.72 (0.96–3.08)	1.01 (0.68–1.49)	**1.94 (1.08–3.46)**	1.03 (0.70–1.55)
***Sex***						
Male	1	1	1	1		
Female	1.31 (0.96–1.8)	1.29 (1.04–1.61)	1.28 (0.91–1.80)	1.29 (1.01–1.65)	1.26 (0.89–1.79)	1.28 (1.0–1.63)
***Educational level***						
No formal education	1	1	1	1	1	1
Primary completed or less	2.26 (1.54–3.33)	1.16 (0.88–1.54)	1.96 (1.29–2.97)	0.97 (0.73–1.27)	**1.87 (1.22–2.87)**	0.94 (0.71–1.25)
Secondary school completed	4.53 (2.03–10.11)	2.44 (1.32–4.52)	3.01 (1.42–6.41)	1.7 (0.89–3.23)	**2.91 (1.36–6.26)**	1.74 (0.92–3.28)
High school and above	2.95 (1.86–4.69)	1.39 (1.03–1.88)	2.01 (1.21–3.36)	0.84 (0.60–1.18)	**1.91 (1.13–3.22)**	0.84 (0.60–1.19)
***Residence***						
Rural			1	1	1	1
Urban			1.49 (0.92–2.43)	1.34 (0.91–1.96)	1.45 (0.89–2.36)	1.34 (0.91–1.98)
***Wealth quintile***						
Q_1_ (lowest)			1	1	1	1
Q_2_			1.58 (0.94–2.65)	1.28 (0.91–1.80)	1.50 (0.88–2.53)	1.27 (0.89–1.81)
Q_3_			1.67 (1.05–2.65)	1.31 (0.90–1.92)	**1.61 (1.01–2.57)**	1.33 (0.91–1.96)
Q_4_			2.12 (1.29–3.48)	1.89 (1.33–2.69)	**1.98 (1.21–3.23)**	**1.84 (1.28–2.64)**
Q_5_ (Highest)			4.41 (2.63–7.41)	2.43 (1.55–3.82)	**4.03 (2.41–6.73)**	**2.38 (1.50–3.77)**
***Health insurance***						
No			1	1	1	1
Yes			1.24 (0.88–1.75)	1.98 (1.51–2.59)	1.22 (0.87–1.73)	**1.97 (1.51–2.57**)
***Type of job employment***						
Public sector			1	1	1	1
Private sector			0.97 (0.39–2.37)	0.66 (0.34–1.28)	0.97 (0.40–2.36)	0.66 (0.34–1.29)
Self-employed			1.61 (0.90–2.90)	0.66 (0.44–1.0)	1.66 (0.93–2.97)	0.68 (0.45–1.02)
Informal employment			1.68 (0.66–4.26)	1.12 (0.61–2.08)	1.82 (0.70–4.75)	1.21 (0.65–2.27)
***Chronic health conditions***						
None					1	1
One chronic condition					1.13 (0.80–1.59)	0.85 (0.66–1.10)
Two or more chronic conditions					**2.04 (1.29–3.22)**	**1.39 (1.01–1.92)**
***Self-rated health***						
Good/very good					1	1
Moderate					0.67 (0.49–0.93)	0.89 (0.68–1.17)
Bad/very bad					0.50 (0.31–0.79)	0.75 (0.55–1.01)

Note: ^a^Other healthcare facility includes charity clinic, charity hospital, home visit and other.

The odds of private facility utilization vs. other providers were significantly higher with increasing wealth quintile. Compared with the lowest quintile, the highest quintile was four times more likely to use private relative to other services (OR 4.03; 95% CI 2.41–6.73). Respondents with two or more chronic conditions had higher odds of using private vs. other services (OR 2.04; 95% CI 2.19–3.22) compared with those reporting no chronic health conditions.

Regarding public outpatient care service utilization, the odds of using public vs. other facilities were higher among the rich and richest wealth quintiles compared with the poorest quintile. Older adults who had health insurance coverage were two times more likely to use a public facility and those with multimorbidity were 39% more likely to use public relative to other facility types.

### Health system responsiveness

The degree of responsiveness was estimated by the percentage of ‘good/very good’, ‘moderate’ and ‘bad/very bad’ answers as indicated in Table 3. For each of the seven domains of health system responsiveness, the most common response was ‘good/very good’. A significantly larger proportion of respondents who visited a private for-profit outpatient facility rated the time waited before being attended to as ‘good/very good’ (*p* < 0.001) than among those who received care in public facilities. Similarly, a large proportion of respondents reported their experience of being treated respectfully as ‘good/very good’ while visiting private for-profit outpatient care compared to a private facility (*p* = 0.0004). Given similar significant results for private for-profit facilities as ‘good/very good’ for each domain, a significantly higher proportion of respondents reported ‘good/very good’ cleanliness of private for-profit healthcare facilities (*p* < 0.001).

**Table 3.** Health system responsiveness among older adults who received outpatient healthcare in the past 12 months, by type of facility, SAGE Ghana Wave 1, 2007/08.

	Private for-profit		Private not-for-profit		Public services			
	n	%	n	%	n	%	F	*p*
For the last visit to a healthcare provider rate the following:								
The amount of time waited before being attended to:								
	(n = 396)		(n = 174)		(n = 1306)			
Very good/good	288	74.1	76	45.1	782	60.7	7.7	< 0.0001
Moderate	81	20.2	57	33.5	342	24.7		
Bad/very bad	26	5.7	41	21.4	182	14.6		
Your experience of being treated respectfully:								
	(n = 397)		(n = 175)		(n = 1325)			
Very good/good	335	89.4	124	71.6	1012	78.3	5.4	0.0004
Moderate	39	9.9	47	25.9	269	19.6		
Bad/very bad	3	0.7	4	2.5	25	2.1		
How clearly healthcare providers explained things to you?								
	(n = 397)		(n = 174)		(n = 1304)			
Very good/good	326	82.1	104	61.1	894	68.9	5.9	0.0003
Moderate	56	14.9	54	29.9	308	22.8		
Bad/very bad	15	3.0	16	9.0	102	8.3		
Your experience in being involved in making decisions for your treatment:								
	(n = 397)		(n = 174)		(n = 1303)			
Very good/good	291	73.4	90	54.7	806	60.9	8.7	0.0009
Moderate	81	20.1	50	27.9	317	24.1	5.0	
Bad/very bad	25	6.5	34	17.4	180	14.9		
The way the health services ensured you could talk privately to health providers:								
	(n = 397)		(n = 1306)		(n = 175)			
Very good/good	350	88.0	126	72.4	1033	77.7	3.9	0.0091
Moderate	44	11.5	44	25	239	18.7		
Bad/very bad	3	0.5	5	2.6	34	3.6		
…the ease with which you could see a healthcare provider you were happy with?								
	(n = 397)		(n = 175)		(n = 1307)			
Very good/good	296	73.8	88	49.7	808	60	6.7	< 0.0001
Moderate	86	22.8	68	37.7	376	28.1		
Bad/very bad	15	3.4	19	12.6	123	11.9		
…the cleanliness in the health facility?								
	(n = 397)		(n = 174)		(n = 1323)			
Very good/good	376	95.8	153	89	1124	85.5	7.1	< 0.0001
Moderate	20	4.0	16	8.4	153	11.9		
Bad/very bad	1	0.2	5	2.6	25	2.6		

Notes: n: unweighted; percentage: weighted. Private not-for-profit: charity clinics and hospitals. The highest numbers in the column are in bold.

## Discussion

The study examined factors associated with the choice of health facilities in outpatient care utilization in Ghana. Public and private outpatient care utilization among older adults aged 50 and older was investigated with respect to predisposing, enabling and need factors based on Andersen’s conceptual framework of healthcare utilization. The results showed that enabling factors (wealth quintile) and need factors (having multiple chronic conditions) were significantly associated with both public and private outpatient care utilization. Among need factors, chronic multimorbidity was positively associated with public and private healthcare utilization. This is in line with previous studies, which reported that having multiple chronic conditions was a strong predictor of healthcare utilization [6,11–13]. Results from a study in India indicated that the prevalence of chronic multimorbidity was high among older adults and utilization of outpatient care increased with an increasing number of chronic diseases [33].

Among enabling factors, the results suggested that wealth quintile is a strong predictor of both public and private outpatient care utilization. Although limited studies about predictors of healthcare utilization distinguish between public and private healthcare facilities, previous studies in LMICs have also reported that wealth status or economic factors were associated with the use of health services [12,15,34]. A study from a high-income country, Hong Kong, suggested that older adults with lower income had significantly greater healthcare needs and were more likely to use both public and private facilities than higher-income groups. However, poor older adults had consulted more governmental facilities and fewer private service providers [10].

The possession of health insurance was positively associated with the use of public health facilities but not with private facilities in our study. Numerous prior studies have noted the importance of health insurance for healthcare utilization [12,35], including one study in Ghana [36]. The availability of health insurance increases healthcare utilization and is one of the means for achieving Universal Healthcare Coverage by removing user fees at the point of use [37]. In this study, of those who reported an outpatient visit in the previous 12 months, respondents who had health insurance were more likely to use public facilities compared to other facilities. Contrary to expectations, health insurance was not a significant predictor for the use of private facilities in outpatient visits. Some private for-profit health facilities do not provide healthcare through the NHIS in Ghana unlike public facilities and charity clinics/hospitals. Financial access to these private for-profit health facilities is a limitation for most of the citizenry and may potentially account for this observation.

Residence (urban/rural) appears not to be a significant factor for the choice of health facilities, suggesting that accessibility of types of health facilities does not differ by residence. This finding contrasts with an earlier study in Uganda which reported that rural residents were more likely to use public health facilities [38]. In the crude analysis (results not shown), location of residence was strongly associated with the use of both private and public facilities. However, the association disappeared following the addition of enabling factors such as wealth quintile and health insurance to the model. There are inconsistent reports about the influence of residence even for total healthcare utilization (private, public and others combined). Some studies highlighted that urban residents are more likely to use healthcare than rural counterparts [16,39], whereas another study in China reported that healthcare utilization has been increased among rural respondents compared to urban residents [40]. Some others have shown that no significant association exists between residence (urban/rural) and healthcare utilization [15,41].

The second question examined was which type of health facility is more responsive in providing healthcare services. Overall, the results suggested that private for-profit health facilities are more responsive than private non-profit and public health facilities. This supports previous study findings in South Africa, which found health system responsiveness was higher in private compared to public outpatient care facilities [28,42,43]. A possible explanation for respondents reporting high percentages of health system responsiveness in each domain for private facilities might be due to differences in trained providers, provider to patient ratios, and good interactions with clients. It is also possible that because private for-profit facilities need to maximize their profit, private facilities are more likely than public facilities to be more responsive in order to attract more clients. A systematic review of public and private healthcare systems’ performance in LMICs suggested that public health facilities lack timeliness and hospitality to patients [44].

In the interpretation of our study results, the following strengths and potential limitations need to be taken into consideration. A major strength of this study was using a nationally representative sample, which enables us to generalize to all older adults residing in Ghana. The use of standardized questionnaires administered by trained interviewers could help to make comparisons with similar data in other LMICs.

However, there are some potential limitations. The first limitation is recall bias, especially for older adults. To minimize the problem of recall of healthcare use, the most recent outpatient visit of health facilities by respondents in the 12 months preceding the survey was analyzed. Secondly, the survey data analysis was based on self-reported data, which is subject to reporting bias. Thirdly, information including contextual and the provider-related factors such as availability of medical services, opening hours and healthcare cost was not included in SAGE. Therefore, adjustments for provider-related variables were not carried out in the analysis although these may explain some of the factors that influence the choice of health facilities. Fourthly, as this study considered the most recent outpatient visit for any health problem, there is no mechanism to know whether they revisited the same facility if needed because some respondents might change health facility or their choice of facility may vary in a subsequent outpatient visit. Finally, due to the cross-sectional nature of the data, causal relationships between predictor variables and utilization of public and private healthcare facilities cannot be established.

## Policy implications

The findings of this study have implications for reducing inequity in access to healthcare, especially in light of the ongoing challenges to improve NHIS coverage [23,45] and efforts to achieve Universal Health Coverage. The results suggest that enabling factors (wealth) were a strong predictor for the use of both public and private health facilities. Although the health insurance scheme in Ghana continues to push to improve access to healthcare, barriers to utilization of both public and private healthcare facilities remain lower for wealthier people. It has been documented in earlier studies that utilization of healthcare in public and private facilities was high among wealthy people even after the abolition of user fees [38,46]. This suggests that some policy changes are needed in Ghana to improve equitable access to healthcare services, especially for poorer older adults, which might take the form of increased subsidies for insurance or addressing mobility for rural older people including the possibility of outreach services for such marginalized groups.

This analysis highlights a high demand for outpatient public and private healthcare services. In this regard, both the public and private sectors need to be prepared to meet the needs of older adults, and the government should also give increased attention to private healthcare facilities. The clear differences in health system responsiveness between public and private providers give the government a set of factors that if addressed would improve quality of healthcare in public health facilities and which might lead to increased utilization and quality of care amongst all groups.

## Conclusion

Results from this study suggest that chronic multimorbidity and higher wealth were factors influencing public and private outpatient care utilization among older adults. Further, the responsiveness of private for-profit health facilities was higher than for public and private not-for-profit health facilities. Future mixed (quantitative and qualitative) method studies could further explore the determinant factors for the choice of health facilities. For example, in qualitative methods, asking both patients and providers about reasons for choosing a particular facility could deepen our understanding of influential factors from both patients’ and providers’ perspectives.

## Paper context

Understanding what drives utilization of health services is critical to achieving Universal Health Coverage. This study examined factors associated with utilization of public and private healthcare services by older adults in Ghana as well as health system responsiveness. Wealth was found to be a major driver of utilization and private services were found to be more responsive than public which identifies areas for policy-makers to build on to improve coverage and quality.
